# Narsoplimab Results in Excellent Survival in Adults and Children With Hematopoietic Cell Transplant Associated Thrombotic Microangiopathy (TA‐TMA)

**DOI:** 10.1002/ajh.70044

**Published:** 2025-08-29

**Authors:** Michelle L. Schoettler, Sunil Kumar Pusarla, Narinder Nangia, Miguel‐Angel Perales, Rafael F. Duarte, Andreas Grauer, Alessandro Rambaldi

**Affiliations:** ^1^ Aflac Cancer and Blood Disorders Center, Children's Healthcare of Atlanta Emory University Atlanta USA; ^2^ Omeros Corporation Seattle Washington USA; ^3^ Adult Bone Marrow Transplantation Service, Department of Medicine Memorial Sloan Kettering Cancer Center New York New York USA; ^4^ Department of Medicine Weill Cornell Medical College New York New York USA; ^5^ Hospital Universitario Puerta de Hierro Majadahonda Madrid Spain; ^6^ Department of Oncology and Hematology University of Milan and Azienda Socio‐Sanitaria Territoriale Papa Giovanni XXIII^,^Piazza OMS Bergamo Italy

**Keywords:** hematopoietic cell transplantation, mortality, narsoplimab, transplant associated thrombotic microangiopathy

## Abstract

Inappropriate complement activation is a key driver of hematopoietic cell transplant‐associated thrombotic microangiopathy (TA‐TMA). Treatment with narsoplimab, an inhibitor of MASP‐2, the effector enzyme of the lectin pathway, resulted in a response rate of 61% in a Phase 2 clinical trial in adults with TA‐TMA. Given these promising results, a global expanded access program (EAP) was established facilitating compassionate use treatment. Herein, we report the survival of children (< 16 years old) and adults (≥ 16 years old) enrolled in the EAP from October 2017 to October 2023 (*n* = 136). Among children (*n* = 50), the majority underwent allogeneic HCT (*n* = 44); 37 were considered high‐risk (HR) and 30 (81.1%) had organ dysfunction at the time of TA‐TMA diagnosis. One‐year overall survival (OS) in pediatric allogeneic recipients with HR TA‐TMA who received narsoplimab as first‐line therapy (*n* = 12) was 75.0% and 56.2% in those who received treatment as ≥ second‐line (*n* = 25, 20 refractory to eculizumab). One‐year OS in the six children with a solid tumor who underwent an autologous HCT was 80%. Among the 86 adults, 84 received an allogeneic HCT, 65 had HR TA‐TMA and, among them, 57 (87.7%) had organ dysfunction at diagnosis. One‐year OS in allogeneic adults with HR TA‐TMA who received narsoplimab as first line therapy (*n* = 49) was 58.0%, and 40.5% in those who received narsoplimab as ≥ second line therapy (*n* = 16). There were no concerning safety signals. In this real‐world study enriched with patients with severe TA‐TMA, survival was excellent in children and adults, supporting the use of narsoplimab.

## Graphical abstract

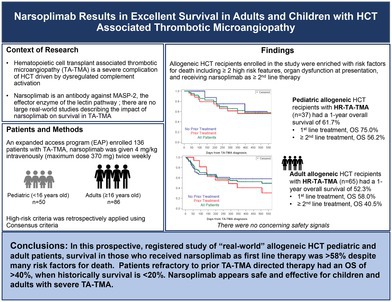

## 1 Introduction

Transplant‐associated thrombotic microangiopathy (TA‐TMA) is a common complication of hematopoietic cell transplantation, impacting 10%–30% of allogeneic recipients in contemporary studies [1, 2, 3, 4, 5]. In addition to the classic microangiopathic manifestations of TMA, consumptive anemia and thrombocytopenia, in the HCT setting, 50%–80% develop a severe phenotype defined as multiorgan dysfunction (MOD) [3, 6, 7, 8]. Historically, mortality rates of severe TA‐TMA exceed 80% [6, 9, 10]. Endothelial damage after multiple recurrent hits and subsequent complement dysregulation are thought to be the primary drivers of TA‐TMA [11, 12, 13, 14, 15, 16]. Prior studies have demonstrated evidence of alternative, classical, lectin, and terminal pathway activation in patients with TA‐TMA [14, 17, 18, 19, 20, 21, 22, 23].

The lectin pathway is activated by pattern recognition molecules in the setting of damaged tissue (damage‐associated molecular patterns, DAMPs) or microbes (pathogen‐associated molecular patterns) [24, 25]. MASP‐2 is the effector enzyme of the lectin pathway and cleaves C4 and C2 to form C3 convertase. C3 convertase splits C3 into C3a and C3b, generating potent anaphylatoxins (C3b and subsequently C5b), which result in additional endothelial damage and, ultimately, terminal complement activation resulting in the formation of the membrane attack complex (MAC, measured in the blood as sC5b‐9). A key non‐canonical function of LP activation is crosstalk with the coagulation cascade; in mouse models, microfluidic devices, and human studies, LP activation promotes a prothrombotic milieu, which is also thought to contribute to TA‐TMA pathogenesis [26, 27, 28].

Narsoplimab is a human, monoclonal antibody to MASP‐2. In a pivotal Phase 2 study, narsoplimab treatment in adults with TA‐TMA (*n* = 28) resulted in a response rate of 61% and median survival of 274 days [29]. Given these promising results, the company sponsored a global expanded access program (EAP), facilitating compassionate‐use treatment of narsoplimab for children and adults with TA‐TMA. While several case reports and series have described outcomes of those treated with narsoplimab via the EAP [30, 31, 32], a comprehensive analysis of survival of the entire EAP cohort has yet to be published. The objective of this study was to describe the survival of adult and pediatric EAP‐enrolled participants.

## 2 Methods

All expanded access program (EAP) recipients treated for TA‐TMA from October 15, 2017 to October 16, 2023 with narsoplimab and a known date of death or last follow‐up were included in this study (clinicaltrials.gov NCT04247906). Pediatric recipients were defined as those aged < 16 years old at enrollment, and adults as ≥ 16 years of age. The pediatric age cut‐off was selected to be consistent with an analysis comparing outcomes in adult patients who received narsoplimab as first‐line therapy versus those with severe TA‐TMA who received no TA‐TMA directed therapy. Outcomes of 49 adult patients who received narsoplimab as first‐line therapy are reported both in this study and the comparator study. Inclusion criteria for EAP enrollment included a diagnosis of TA‐TMA defined as thrombocytopenia and evidence of microangiopathy. Exclusion criteria included malignant hypertension or uncontrolled infection. There were no performance status or organ function requirements to enroll in the study. The recommended dosing in the EAP is 4 mg/kg for patients < 50 kg and 370 mg for patients ≥ 50 kg administered IV twice weekly initially for at least 8 weeks; change in dosing frequency was allowed based on the treating physician's assessment of treatment response. All patients or their legal guardians signed informed consent, and treatment was approved by the respective institutional IRB.

Consensus risk stratification criteria were retrospectively assigned to the cohort [33]. If a patient had any of the following features at TA‐TMA diagnosis, they were considered high‐risk: sC5b‐9 > upper limit of normal (ULN), random urine protein to creatinine ratio (rUPCR) of ≥ 1 mg/mg, Grade 2–4 acute graft‐versus‐host disease (GVHD) prior to or at the time of TA‐TMA diagnosis, organ dysfunction as previously defined, a lactate dehydrogenase > 2X the ULN, or a bacterial or fungal infection [33]. Graft‐versus‐host disease (GVHD) was staged and graded using MAGIC criteria [34].

Descriptive statistics were used to summarize the cohort. The estimated overall survival was calculated from the time of TA‐TMA diagnosis, and patients were censored until the day of death or last known follow‐up. Patients were analyzed by age group: pediatric and adult. Within each age group, patients were stratified into two groups: those who received narsoplimab as first‐line medication and those who received narsoplimab as second‐line medication. SAS 9.4 (Cary, NC) was used to complete the analysis.

## 3 Results

**FIGURE 1:**
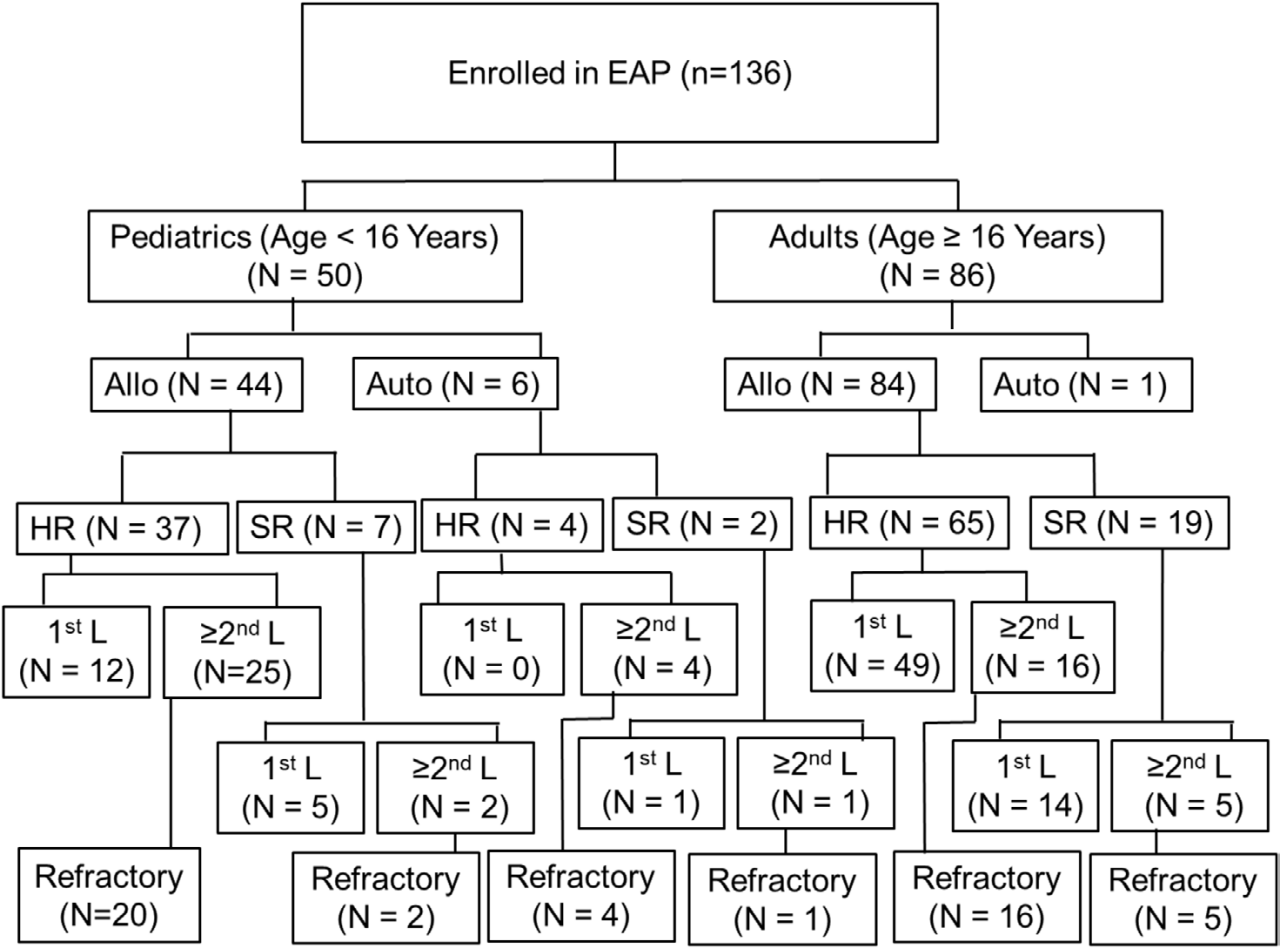
Patients enrolled in the EAP. Abbreviations: allogeneic (allo), autologous (auto), emergency access program (EAP), high‐risk (HR), standard‐risk (SR). Among adults, there was one patient with missing HCT type (allogeneic versus autologous) information.

A total of 136 patients were enrolled in the study; 86 adults and 50 children (Figure 1). Patients were enrolled worldwide over 28 centers, with the majority enrolled in the United States of America (*n* = 60, 44.1%), Italy (*n* = 36, 26.5%), and India (*n* = 20, 14.7%), Table S1.

### 3.1 Pediatric Cohort, TA‐TMA Manifestations, Risk Stratification and Severity

**FIGURE 2:**
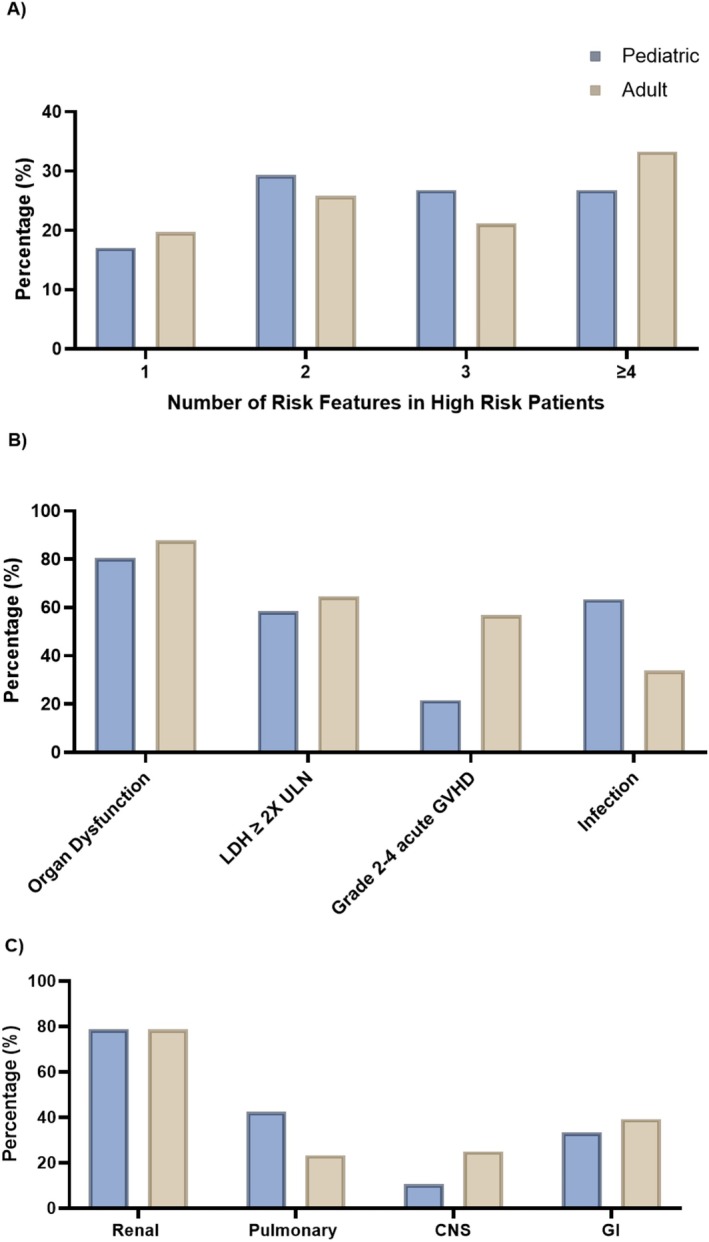
Number of high‐risk features (A), frequency of each high‐risk feature (B), and organ manifestations of those with high‐risk TA‐TMA (C) in pediatric and adult HCT recipients. The percentage and frequency of risk factors among patients who met high‐risk criteria are depicted by age group; most children and adults had ≥ 2 risk factors (A). The percentage and frequency of each high‐risk feature are depicted by age among high‐risk patients: most children and adults had organ dysfunction at the time of TA‐TMA diagnosis (B). The percentage and frequency of organ manifestations among the patients with organ dysfunction at TA‐TMA diagnosis are depicted by age; renal manifestations were most common in children and adults, followed by pulmonary manifestations in children and GI in adults (C). [Color figure can be viewed at wileyonlinelibrary.com]

Among the 50 pediatric patients enrolled, the median age of participants was 8 years (range 0 to 15 years), 24 (48%) were male, and 36 (72%) received a myeloablative regimen. TA‐TMA was diagnosed a median of 98 days (range 0 to 460) post HCT (Table 1). The most common HCT indications were hematologic malignancy (*n* = 18, 36%), non‐malignant blood disorder (*n* = 9, 18%), and solid tumor (*n* = 6, 12%). Forty‐one (82%) patients met high‐risk criteria: 37 allogeneic recipients and 4 autologous recipients. Among high‐risk patients, most children had ≥ 2 high‐risk features (*n* = 34, 82.9%, Figure 2A); the most common high‐risk feature was organ dysfunction (*n* = 33, 80.5%), followed by concurrent bacterial, viral, or fungal infection (*n* = 26, 63.4%, Figure 2B). Among those with organ dysfunction at diagnosis (severe TA‐TMA), the three most frequently involved organs were the kidneys (*n* = 26, 78.8%), lungs (*n* = 14, 42.4%), and gastrointestinal system (GI, *n* = 11, 33.3%, Figure 2C).

**TABLE 1:** HCT, patient and treatment characteristics.

	Entire cohort (*n* = 136) *N* (%)	Pediatric recipients (*n* = 50) *N* (%)	Adult recipients (*n* = 86) *N* (%)
Age at HCT (years, median, range)	22.4 (0, 72)	8 (0, 15.6)	48.8 (16, 72)
Sex			
Female	66 (48.5)	26 (52.0)	40 (46.5)
Male	70 (51.5)	24 (48.0)	46 (53.5)
HCT indication			
HM	55 (40.4)	18 (36)	37 (43.0)
NMH	14 (10.3)	9 (18)	5 (5.8)
Immune def/dysregulation	7 (5.1)	1 (2)	6 (7.0)
Solid tumor	6 (4.4)	6 (12)	0 (0.0)
Other	2 (1.5)	1 (2)	1 (1.2)
Missing	52 (38.2)	15 (30)	37 (43.0)
Preparative intensity			
Myeloablative	63 (46.3)	36 (72.0)	27 (31.4)
RIC	35 (25.7)	5 (10.0)	30 (34.9)
NMA	9 (6.6)	1 (2.0)	8 (9.3)
Missing	29 (21.3)	8 (16.0)	21 (24.4)
HCT type			
Allogeneic	128 (94.1)	44 (88.0)	84 (97.7)
Autologous	7 (5.1)	6 (12.0)	1 (1.2)
Missing	1 (0.07)	0 (0)	1 (1.2)
Stem cell source^a^			
BMT	29 (22.7)	19 (43.2)	10 (11.9)
PBSC	76 (59.4)	20 (45.5)	56 (66.7)
UC	3 (2.3)	1 (2.3)	2 (2.4)
Missing	20 (15.6)	4 (9.1)	16 (19.0)
Donor^a^			
Related	41 (32.0)	16 (36.4)	25 (29.8)
Unrelated	39 (30.5)	12 (27.3)	27 (32.1)
Missing	48 (37.5)	16 (36.4)	32 (38.1)
Maximum acute GVHD grade^a^			
Grade 0–1	60 (46.9)	28 (63.6)	32 (38.1)
Grade 2–4	41 (32.0)	8 (18.2)	33 (39.3)
Missing	27 (21.1)	8 (18.2)	19 (22.6)
Day of TA‐TMA diagnosis (median, range)	88 (0, 1042)	98 (0, 460)	85 (0, 1042)
Received prior TA‐TMA directed therapy	53 (39.0)	32 (64)	21 (24.4)
Prior medications received			
Eculizumab	50 (36.8)	31 (62.0)	19 (22.1)
Ravulizumab	2 (1.5)	2 (4.0)	0
Defibrotide	8 (5.9)	6 (12.0)	2 (2.3)
Prior PLEX	24 (17.6)	7 (14.0)	17 (19.8)
Number of narsoplimab doses (median, range)	9 (1, 170)	13 (1, 170)	7 (1, 75)
Duration of narsoplimab therapy (weeks, median, range)	5.71 (0.1, 92)	7.5 (0.1, 92)	4.43 (0.1, 36.9)
Duration of narsoplimab therapy			
< 1 week	16 (11.8)	3 (6.0)	13 (15.1)
≥ 1 week to < 4 weeks	39 (28.7)	11 (22.0)	28 (32.6)
≥ 4 weeks to < 8 weeks	38 (27.9)	15 (30.0)	23 (26.7)
≥ 8 weeks	42 (30.9)	21 (42.0)	21 (24.4)
Missing	1 (0.7)	0 (0)	1 (1.2)
Day from TA‐TMA diagnosis to first dose of narsoplimab (median, range)^b^	30 (−17, 421)	34 (0, 421)	26.5 (−17, 309)

Abbreviations: BMT, bone marrow transplant; GVHD, graft versus host disease; HCT, hematopoietic cell transplant; HM; hematologic malignancy; immune def/dys, immune deficiency/dysregulation; NMA, non‐myeloablative; NMH, non‐malignant hematologic blood disorder; PBSC, peripheral blood stem cell; PLEX, plasma exchange therapy; RIC, reduced intensity conditioning; TA‐TMA; transplant associated thrombotic microangiopathy; UC, umbilical cord.

^a^ Allogeneic HCT recipients only.

^b^ Day‐17 is likely a data entry error, but could not be confirmed at the time of submission.

### 3.2 Pediatric Cohort, Prior TA‐TMA Directed Therapies

**FIGURE 3:**
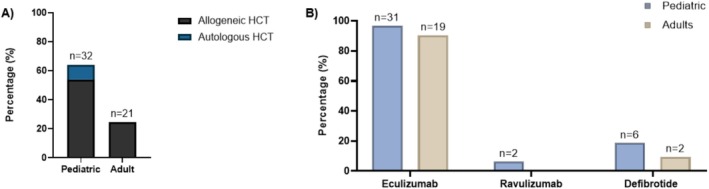
Proportion of patients who received narsoplimab as ≥ second‐line therapy in pediatric and adults by HCT type (A) and prior treatments received (B). Sixty‐four percent of children (*n* = 32) received narsoplimab as ≥ second‐line therapy for TA‐TMA; the majority were allogeneic recipients, while only 24.4% of adults (*n* = 21) received narsoplimab as ≥ second‐line therapy (A). Most children (*n* = 31, 96.9%) and adults (*n* = 19, 90.4%) were treated with eculizumab prior to narsoplimab (B). [Color figure can be viewed at wileyonlinelibrary.com]

Thirty‐two (64%) enrolled children received one or more TA‐TMA directed medication prior to receiving narsoplimab; 27 allogeneic recipients and five autologous recipients (Figure 3A). Among those who received ≥ 1 prior treatment, most received eculizumab (*n* = 31, 96.9%, Figure 3B) and 27 (84.4%) received narsoplimab because the provider deemed them refractory to prior medication(s).

### 3.3 Pediatric Cohort, Narsoplimab Dosing

Pediatric patients received a median of 13 doses of narsoplimab on study (range 1, 170) over a median duration of 7.5 weeks (range 0.1, 92). Three patients received less than 1 week of therapy; all died within 1 week before they could receive additional treatment. Eleven (22%) received ≥ 1 week of therapy and < 4 weeks, 15 (30.0%) ≥ 4 weeks and < 8 weeks, and 21 (42%) ≥ 8 weeks of therapy. The first dose of narsoplimab was given a median of 34 days after TA‐TMA diagnosis (range 0, 421). Among those who received narsoplimab as first‐line therapy, the median time from TA‐TMA diagnosis to first dose was 21.5 days (range 0, 208) compared to 46 days (range 10, 421) in those who received narsoplimab as ≥ second‐line therapy (Table 1).

### 3.4 Pediatric Cohort, Allogeneic HCT Recipient Overall Survival

Most enrolled pediatric patients received an allogeneic HCT (*n* = 44, 88%). Among them, the most common stem cell source was peripheral blood stem cells (*n* = 20, 45.5%) and 16 (36.4%) received a related donor graft. Among all allogeneic recipients, the estimated 1‐year OS from TA‐TMA diagnosis was 53.2% (95% CI: 37.0, 69.4). Twenty‐seven (61.4%) received narsoplimab as a ≥ second‐line agent; one‐year OS was 51.7% (95% CI: 31.8, 71.6) in previously treated patients versus 58.3% (95% CI: 31.3, 85.2) in those who received narsoplimab as a first line agent (*n* = 17).

### 3.5 Pediatric Cohort, High‐Risk Allogeneic HCT Recipients Overall Survival

**FIGURE 4:**
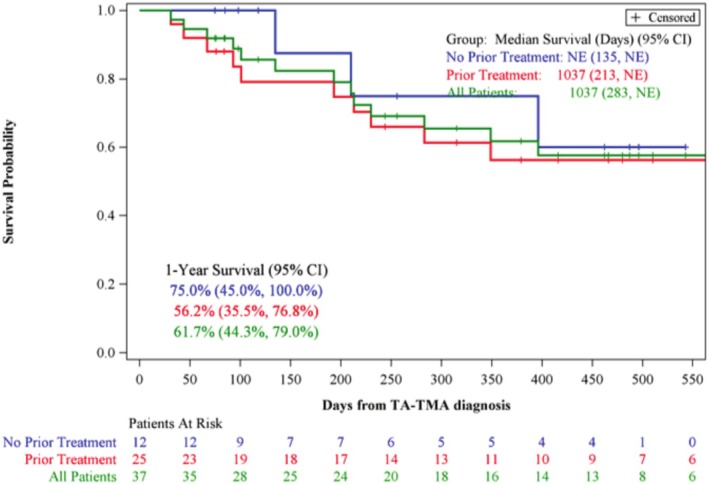
Overall survival in pediatric allogeneic recipients with high‐risk TA‐TMA (*n* = 37) stratified by first and ≥ second‐line therapy. OS all pediatric high‐risk allogeneic recipients (*n* = 37) stratified by narsoplimab given first or ≥ second‐line therapy (B). [Color figure can be viewed at wileyonlinelibrary.com]

Thirty‐seven (84.1%) allogeneic HCT recipients had high‐risk TA‐TMA, and 1‐year OS of all high‐risk TA‐TMA children was 61.7% (95% CI: 44.3, 79.0). One‐year OS in those who received narsoplimab as second‐line or more (*n* = 25, 67.6%) was 56.2% (95% CI: 35.5, 76.8). The estimated one‐year OS in those who received narsoplimab as first‐line therapy (*n* = 12, 32.4%) was 75.0% (95% CI: 45.0, 100, Figure 4).

### 3.6 Pediatric Cohort, Autologous HCT Recipients

Six autologous HCT recipients were enrolled in the EAP. The median age was 5.1 years (range 2.7, 6.1), 3 (50%) were female, and all received an HCT for a solid tumor. Data on the number of transplants (1st, 2nd, or 3rd) prior to the development of TA‐TMA is unavailable. The median day of TA‐TMA diagnosis was 44 days post HCT, and 4 met high‐risk criteria. Five received prior TA‐TMA‐directed therapy, and all of them were known to be refractory to prior treatment. The estimated one‐year OS among autologous recipients was 80% (95% CI: 44.9, 100.0, Figure S1).

### 3.7 Adult Cohort, TA‐TMA Manifestations and Risk Stratification

Among the 86 enrolled adult HCT recipients, the median age was 48.8 years (range 16–72 years), 46 (53.5%) were male, and 27 (31.4%) underwent myeloablative conditioning. The median day of TA‐TMA diagnosis post HCT was 85 days (range 0 to 1042). Sixty‐six patients (76.7%) were classified as high‐risk, 65 allogeneic recipients and one autologous recipient. Among them, 80.3% had ≥ 2 high‐risk features present (Figure 2A). The most common high‐risk feature was organ dysfunction, which was present at diagnosis in 58 (87.9%) adults with high‐risk TA‐TMA (Figure 2B). Among adults with organ manifestations, the three most frequently involved organs were the kidney (*n* = 44, 75.9%), GI system (*n* = 22, 37.9%), and central nervous system (*n* = 14, 24.1%, Figure 2C). Most adults underwent an allogeneic HCT (*n* = 84), one received an autologous HCT, and one adult had HCT type missing.

### 3.8 Adult Cohort, Prior TA‐TMA Directed Therapies

Twenty‐one (24.4%) enrolled adults received one or more TA‐TMA‐directed medications prior to receiving narsoplimab; 16 allogeneic recipients with high‐risk TA‐TMA and 5 with standard‐risk TA‐TMA (Figure 3A). Prior treatments included eculizumab (*n* = 19, 22.4%), ravulizumab (*n* = 0, 0%), and defibrotide (*n* = 2, 2%, Figure 3B). Of those treated with narsoplimab as second‐line therapy, 21 received narsoplimab because the provider deemed them refractory to prior medication(s).

### 3.9 Adult Cohort, Narsoplimab Dosing

Adults received a median of seven doses of narsoplimab (range 1–75) over a duration of a median of 4.4 weeks (range 0.1–36.9 weeks). Thirteen enrolled patients (15.1%) received less than 1 week of narsoplimab. Twenty‐eight (32.5%) received ≥ 1 and < 4 weeks of therapy, 23 (26.7%) received ≥ 4 weeks to < 8 weeks of therapy, and 21 (24.14%) received ≥ 8 weeks of therapy. The first dose of narsoplimab was given a median of 26.5 days after TA‐TMA diagnosis (range 17–309). Notably, we assume receiving treatment 17 days prior to TA‐TMA diagnosis is a data entry error; however, no clarification from the center was received to correct the data. Among those who received narsoplimab as first‐line therapy, the median time from TA‐TMA diagnosis to first dose was 15 days (range 17 to 206) compared to 67 days (range 12–309) in those who received narsoplimab as ≥ second‐line therapy.

### 3.10 Adult Cohort, Allogeneic HCT Recipient Overall Survival

Most enrolled adults received an allogeneic HCT (*n* = 84, 97.7%); among them, the most common stem cell source was PBSC (*n* = 56, 66.7%) and 27 (32.1%) received an unrelated donor graft. The most frequent HCT indications included an underlying diagnosis of hematologic malignancy (*n* = 37, 43%) and immune deficiency/dysregulation (*n* = 6, 7.0%). Among all adult allogeneic HCT recipients, the estimated one‐year OS from TA‐TMA diagnosis was 49.5% (95% CI: 37.6, 69.4). One‐year OS in the 27 (34.5%) who received narsoplimab as ≥ second‐line therapy was 42.7% (95% CI: 19.7, 65.8). Estimated 1‐year OS in the 63 (75%) adults who received narsoplimab as the front‐line agent was 52.7% (95% CI: 38.9, 66.4). In a sensitivity model excluding the seven allogeneic HCT recipients who died within 1 week of receiving their first dose of narsoplimab, the 1‐year OS was 56.4% (95% CI: 42.2, 70.6).

### 3.11 Adult Cohort, High‐Risk Allogeneic HCT Recipients Overall Survival

**FIGURE 5:**
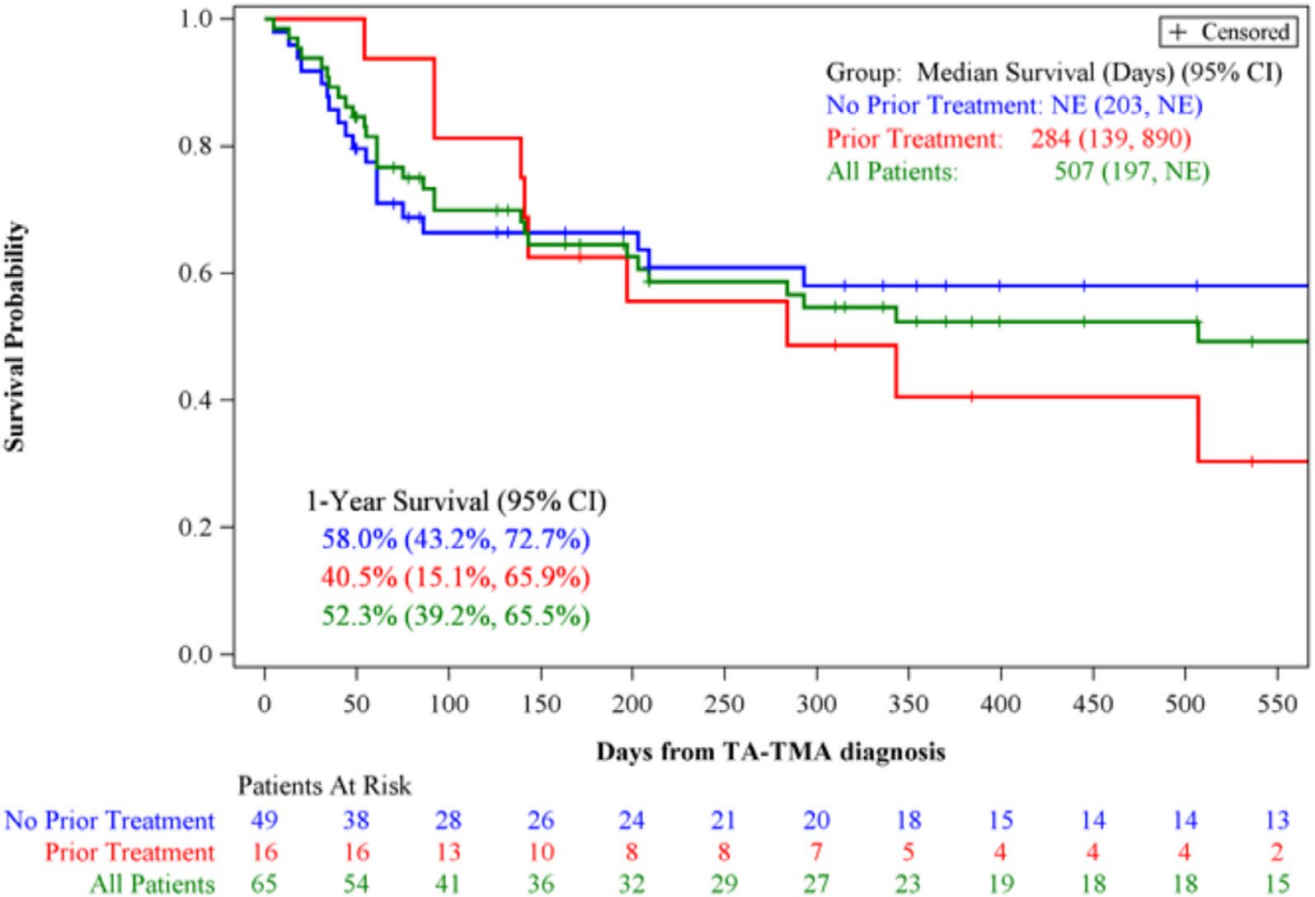
Overall survival in adult allogeneic recipients with high‐risk TA‐TMA treated with narsoplimab stratified by first and ≥ second‐line therapy. Overall survival of all allogeneic adult HCT recipients (*n* = 83) stratified by narsoplimab given first or ≥ second‐line (A), and OS of all high‐risk allogeneic HCT recipients (*n* = 65) stratified by narsoplimab given first‐ or ≥ second‐line (B). One patient is missing TA‐TMA diagnosis data and is excluded. [Color figure can be viewed at wileyonlinelibrary.com]

Among all adult high‐risk TA‐TMA allogeneic recipients (*n* = 65), the one‐year OS was 52.3% (95% CI: 39.2, 65.5). Survival in those who received narsoplimab as ≥ second‐line therapy (*n* = 16, 24.6%) was 40.5% (95% CI: 15.1, 65.9) and 58.0% (95% CI: 43.2, 72.7) in those who received narsoplimab as first‐line therapy (*n* = 49, 75.4%), Figure 5. Given that only one adult recipient received an autologous HCT, estimated 1‐year survival was not calculated. That individual died 49 days after TA‐TMA diagnosis.

### 3.12 Safety‐All Patients

There were 113 serious adverse events (SAE) reported in 54 participants. Events reported ≥ 5 times within an organ system and individual events which occurred in ≥ 2% of the population are summarized in Table 2. Narsoplimab was well tolerated. The SAEs reported in > 10% of patients included infections (29 events, 18 patients) and respiratory/mediastinal complications (20 events, 19 patients). Three patients developed meningitis; the organism was reported in one case (enterococcus), but organisms were unknown in the other two cases. All three patients received C5 inhibition prior to narsoplimab.

**TABLE 2:** Reported adverse events by organ class in organ classes with ≥ 5 events.

Serious adverse events—adults + pediatric	Events	Patients
System organ class		
Preferred term		
Any	113	54
Blood and lymphatic system disorders	8	7
Febrile neutropenia	5	5
General disorders and administration site conditions	11	11
Death	3	3
Multiple organ dysfunction syndrome	4	4
Infections and infestations	29	18
Meningitis	3	3
Sepsis	7	6
Septic shock	5	5
Nervous system disorders	7	7
Posterior reversible encephalopathy syndrome	3	3
Renal and urinary disorders	9	8
Acute kidney injury	3	3
Respiratory, thoracic and mediastinal disorders	20	19
Respiratory failure	9	9
Vascular disorders	6	5
Hypotension	3	2

*Note*: Reported adverse events (AE) with ≥ 5 events with the organ system. Individual AEs are reported if they occurred in more than 2% of the enrolled participants.

## 4 Discussion

In a large cohort of patients with TA‐TMA enriched with risk factors for death including high‐risk features, particularly organ dysfunction, refractory TA‐TMA, and a delay in TA‐TMA‐directed therapy [3, 35], treatment with narsoplimab via an EAP resulted in a one‐year overall survival in allogeneic HCT adult and pediatric recipients of 49.5% and 53.2%, respectively. In a sensitivity analysis excluding seven adults who died after receiving only one dose of narsoplimab, the 1‐year OS of adult allogeneic recipients was 56.4%. Further, there were no significant safety signals.

Given that this is a single‐arm study without a comparison arm, placing these results in the context of the current literature has some challenges. Prior studies reporting survival in TA‐TMA are highly heterogenous; variations include TA‐TMA diagnostic criteria, inclusion of HCT type (allogeneic only or allogeneic and autologous), variations in high‐risk stratification, differences in proportions of severe TA‐TMA (i.e., organ dysfunction/failure), and the reported time of survival (from HCT day 0 or from TA‐TMA diagnosis)—all are known to impact OS [7, 36, 37, 38, 39, 40]. An international consensus group recently proposed a novel risk stratification with the purpose of identifying patients at highest risk of dying *before* they developed significant organ dysfunction, which is challenging to reverse [33]. Emerging data suggest that survival rates in patients defined as high‐risk using this definition have varied [9, 37, 40]. However, in both contemporary and historic studies, mortality remains high after allogeneic HCT in TA‐TMA patients with organ dysfunction regardless of the diagnostic or risk stratification criteria utilized [37, 41]. Outcomes of patients with lung, central nervous system, and gastrointestinal TA‐TMA manifestations are very poor, and both children and adults in this cohort are enriched with these severe features [35, 42]. Finally, mortality rates in those refractory to upfront TA‐TMA‐directed therapies, that is, C5 inhibition, have particularly dismal survival (< 20%), suggesting that outcomes in these patients should be considered separately from those who receive narsoplimab as upfront therapy [3, 42].

When comparing the survival of patients in this study to available literature, the most appropriate comparison groups are cohorts of allogeneic HCT recipients with TA‐TMA and a similar proportion of organ dysfunction, or severe disease. In adult cohorts with TA‐TMA and organ dysfunction treated with supportive care ± plasma exchange therapy, 1‐year OS in similar cohorts ranged from 0% to 27% [36, 38, 39, 41, 43, 44, 45]. There are fewer available data in similar untreated pediatric cohorts, though in a historic cohort of 12 allogeneic pediatric recipients with TA‐TMA and organ dysfunction, 1‐year OS was 18% [6]. Thus, 1‐year survival in high‐risk, severe, pediatric and adult TA‐TMA allogeneic HCT participants treated with narsoplimab in the EAP, 61.7% and 52.3% respectively, appears clinically significant and supports the use of narsoplimab for severe TA‐TMA. While high‐risk data were not available on all patients, the relatively similar survival in those with known high‐risk features including organ dysfunction and those with missing data suggests that the EAP cohort was enriched with severe disease. Additional studies statistically comparing a similar cohort are warranted.

While patients with ongoing TA‐TMA and organ dysfunction are thought to be more difficult to salvage, there are limited data on survival among those refractory to upfront therapy, particularly in those who then receive a second TA‐TMA directed therapy. In two multi‐institutional studies, 1‐year OS in children with no response to eculizumab was 0% (*n* = 7) [42] and 28.4% (*n* = 73) [46]. In another multi‐institutional study, all children with no response to eculizumab died (*n* = 43) [47]. While additional studies are needed, these data suggest that survival is very poor in refractory patients. The 1‐year OS in HR children and adults who received narsoplimab as ≥ second‐line therapy was 56.2% and 40.5%, respectively, and 84% of children and 100% of adults who received narsoplimab as ≥ second‐line therapy were deemed refractory to eculizumab. Again, these survival differences appear clinically significant and suggest that narsoplimab salvaged a meaningful proportion of patients who historically have dismal outcomes.

In this EAP, in addition to a large allogeneic HCT cohort treated with narsoplimab, six children received treatment after an autologous HCT. While this represented a small proportion of the EAP cohort, the reported incidence of TA‐TMA after autologous HCT in children with neuroblastoma is up to 30% [48, 49]. Thus, TA‐TMA treatment in the autologous setting is highly relevant to the pediatric HCT community. Four (66.7%) had high‐risk disease and 5 (83.3%) received narsoplimab as a second‐line agent. The sample size is small, but enriched with severe, refractory disease. Despite this, the 1‐year OS was 80%, again suggesting benefit in this population, though this needs to be studied further.

In addition to the efficacy of narsoplimab and other novel TA‐TMA‐directed therapies, it is imperative to carefully investigate safety. There were no safety signals of concern with narsoplimab treatment in this EAP study. The complement cascade is a key component of the innate immune system and critical in the clearance of bacteria, viruses, and fungi. Terminal complement inhibition (C5) prevents the formation of the membrane attack complement (MAC) and is known to incur a > 2000‐fold increased risk of meningitis [50]. There are emerging data that in the HCT setting, C5 inhibition is associated with an increased risk of non‐meningococcal severe infections [51]. Narsoplimab inhibits only the lectin pathway, allowing the generation of MAC via the alternative and classical pathways [52]. Studies have demonstrated that specific inhibition of the alternative pathway compared to C3 or C5 inhibition results in a preserved ability to clear infections [53, 54]. While there are no specific data on the differential impact of lectin pathway inhibition compared to terminal inhibitors, MASP2 inhibition is not thought to confer additional infectious risks, which may be an important safety issue in the HCT setting when considering TA‐TMA directed therapy. Three patients in this study developed meningitis, but all were treated with C5 inhibition prior to enrollment.

These “real world data” have both strengths and limitations. This is the largest sample size of children and adults who received narsoplimab for TA‐TMA. There were no organ function requirements nor strict exclusion criteria for enrollment. This sample represents the real‐world including patients with very severe disease who likely would not have been eligible for a clinical trial as evidenced by nine dying within 1 week of receiving their first dose. The median 1‐month delay in narsoplimab administration from TA‐TMA diagnosis is likely due to the regulatory requirements of the EAP and was unavoidable in this study. Other studies have suggested that delays in administering TA‐TMA‐directed therapy are associated with an increased risk of death [55, 56]. Despite this, survival in these “real‐world” adults and children with severe TA‐TMA mirrored or exceeded that of the Phase 2 pivotal trial in adults with TA‐TMA [29].

Data were requested from all participating centers, but responses to queries varied and there are missing data. While detailed organ dysfunction information was collected, sC5b‐9 and rUPCR were not obtained on any participants. sC5b‐9 and rUPCR are previously reported important prognostic risk factors in some pediatric cohorts [57]. However, given data supporting the importance of organ dysfunction as a risk factor and the recommendation of the international consensus group to risk stratify in the absence of sC5b‐9 data, this was not considered a major limitation [33]. The data collection forms did not collect some data of interest, including HLA mismatch. No response data are available; it is possible that patients responded to TA‐TMA directed therapy and died of other etiologies. Further, relapse data are unavailable, though relapse may result in death of up to 30% of the malignant population, the predominant indication for HCT in this cohort. However, these limitations bias results against the impact of narsoplimab on severe TA‐TMA; that is, it is plausible that narsoplimab had a more positive impact on non‐relapse related mortality than we can detect with available data, but this does not alter the primary conclusions of the study. As this is not a monitored clinical trial, there is no mechanism to confirm the accuracy of the reported data. Thus, while some unusual outliers were noted, that is, day of TA‐TMA ranges of 0–460 post HCT, outside of confirming the accuracy of the data reported from the center, there were no source documents available to adjudicate data. Finally, treatment approaches and duration were suggested, but there were variations in approach as evidenced by the large range in duration of therapy (0–92 weeks), which adds heterogeneity to the study.

There are currently several recently completed or ongoing clinical trials investigating the safety and efficacy of novel agents for TA‐TMA. Complement C5 inhibition is used off label for TA‐TMA at many institutions, though efficacy and safety data are mixed [3, 6, 9, 35, 42, 47, 51, 58]. Given the current landscape, there is increasing interest in identifying biomarkers and/or clinical features which may predict which agent(s) are more likely to achieve a response in a given patient. Gastrointestinal bleeding is a described risk factor for poor response to eculizumab [35], but there are no other described clinical features or biomarkers to inform a personalized therapeutic approach. sC5b‐9 is elevated in 50%–80% of patients at the time of TA‐TMA diagnosis and is a described prognostic biomarker in multiple pediatric studies. However, a normal sC5b‐9 does not exclude a complement‐mediated pathology, nor is it associated with response in TA‐TMA to C5 inhibition. In related TMAs with known genetic drivers of complement dysregulation, serum complement markers can be normal despite evidence of complement deposition in target organs [59, 60], and complement inhibition strategies are still effective [61]. MASP‐2 is the effector enzyme of the lectin pathway and is located well upstream of the terminal pathway. While sC5b‐9 is a biomarker of terminal pathway activity, it is not a reliable biomarker of lectin pathway activation nor response for MASP‐2 inhibition. There are no known biomarkers predicting response to MASP‐2 inhibition, nor any known complement biomarkers to follow during treatment to assess response [31]. Additional studies addressing this gap are needed.

In conclusion, in this prospective, registered study (clinicaltrials.gov NCT04247906), survival of children and adults with severe TA‐TMA was promising without any concerning safety signals. These data contribute to a growing body of literature suggesting a role for narsoplimab treatment for TA‐TMA [23, 29, 30, 31, 32]. In line with other studies, patients with ongoing severe disease and organ dysfunction were more challenging to salvage, highlighting the importance of early initiation of therapy. Additional prospective studies are needed to confirm these findings and validate efficacy and safety.

## Conflicts of Interest

M.‐A.P. reports honoraria from Adicet, Allogene, Caribou Biosciences, Celgene, Bristol‐Myers Squibb, Equilium, Exevir, ImmPACT Bio, Incyte, Kite/Gilead, Merck, Miltenyi Biotec, MorphoSys, Nektar Therapeutics, Novartis, Omeros, OrcaBio, Pierre Fabre, Sanofi, Syncopation, Takeda, VectivBio AG, and Vor Biopharma; he serves on DSMBs for Cidara Therapeutics and Sellas Life Sciences; he has ownership interests in Omeros and OrcaBio; he has received institutional research support for clinical trials from Allogene, Genmab, Incyte, Kite/Gilead, Miltenyi Biotec, Nektar Therapeutics, and Novartis. R.F.D. reports honoraria and/or institutional research support from Galapagos NV, Gilead Sciences, Janssen, Jazz, Merck Sharp & Dohme, Mundipharma, Omeros, Roche Diagnostics, Sobi, and Takeda. A.R. reports honoraria from Astellas, Pfizer, Amgen, Omeros, Novartis, Kite‐Gilead, Jazz, Celgene‐BMS, Janssen, Roche, Incyte, and AbbVie. M.L.S. is a consultant to Omeros and Alexion. S.K.P., N.N., and A.G. work for Omeros.

## Supporting information


**Data S1:** Supporting Information.

## Data Availability

The data that support the findings of this study are available from the corresponding author upon reasonable request.
