# Supplementary material for: Narsoplimab Results in Excellent Survival in Adults and Children With Hematopoietic Cell Transplant Associated Thrombotic Microangiopathy (TA‐TMA)
Source: Am J Hematol. 2025 Aug 29;100(11):2040–51. doi: 10.1002/ajh.70044 (PMC12516664; doi:10.1002/ajh.70044)
Supplement: Supplementary file 1 — Data S1: Supporting Information. [file AJH-100-2040-s001.docx]

**Supplemental Table 1: Enrollment in the EAP by country and age group**

| Country | Pediatric (n= 50)  N (%) | Adult (n=86)  N (%) |
| --- | --- | --- |
| United States of America | 22 (44) | 38 (44.2) |
| Italy | 15 (30) | 21 (24.4) |
| India | 9 (18) | 11 (12.8) |
| Spain | 1 (2) | 6 (7) |
| United Kingdom | 1 (2) | 4 (4.7) |
| Germany | 1 (2) | 0 (0) |
| Australia | 1 (2) | 0 (0) |
| Netherlands | 0 (0) | 3 (2.5) |
| Belgium | 0 (0) | 2 (2.3) |
| Hong Kong SAR China | 0 (0) | 1 (1.2) |
| Australia | 1 (2) | 0 (0) |

**Supplemental Figure 1: Overall survival of pediatric autologous recipients with TA-TMA treated with narsoplimab**

**
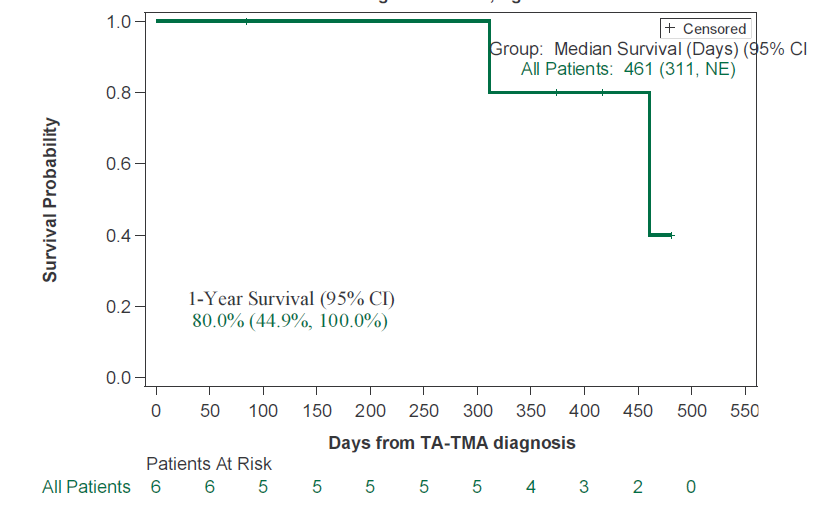
**

Among the 6 pediatric autologous recipients, 1-yr OS was 80.0 (95% CI 44.9,100). While 4 patients had high-risk disease, and 5 received narsoplimab as second-line therapy, given sample size, this cohort was not stratified.
